# Costs and healthcare use of patients with chronic kidney disease in the Northern Territory, Australia

**DOI:** 10.1186/s12913-024-11258-8

**Published:** 2024-07-09

**Authors:** Winnie Chen, Kirsten Howard, Gillian Gorham, Asanga Abeyaratne, Yuejen Zhao, Oyelola Adegboye, Nadarajah Kangaharan, Mohammad Radwanur Rahman Talukder, Sean Taylor, Alan Cass, Territory Kidney Care Steering Committee

**Affiliations:** 1 Menzies School of Health Research, Charles Darwin University, PO Box 41096, Casuarina, Darwin, NT Australia; 2 Menzies Centre for Health Policy and Economics, Faculty of Medicine and Health, University of Sydney, Sydney, Australia; 3 NT Health, Darwin, Australia

**Keywords:** Chronic kidney disease, Chronic diseases, Multimorbidity, Costs, Healthcare use, Health economics, Indigenous health

## Abstract

**Background:**

The burden of chronic kidney disease (CKD) is high in the Northern Territory (NT), Australia. This study aims to describe the healthcare use and associated costs of people at risk of CKD (e.g. acute kidney injury, diabetes, hypertension, and cardiovascular disease) or living with CKD in the NT, from a healthcare funder perspective.

**Methods:**

We included a retrospective cohort of patients at risk of, or living with CKD, on 1 January 2017. Patients on kidney replacement therapy were excluded from the study. Data from the Territory Kidney Care database, encompassing patients from public hospitals and primary health care services across the NT was used to conduct costing. Annual healthcare costs, including hospital, primary health care, medication, and investigation costs were described over a one-year follow-up period. Factors associated with high total annual healthcare costs were identified with a cost prediction model.

**Results:**

Among 37,398 patients included in this study, 23,419 had a risk factor for CKD while 13,979 had CKD (stages 1 to 5, not on kidney replacement therapy). The overall mean (± SD) age was 45 years (± 17), and a large proportion of the study cohort were First Nations people (68%). Common comorbidities in the overall cohort included diabetes (36%), hypertension (32%), and coronary artery disease (11%). Annual healthcare cost was lowest in those at risk of CKD (AUD$7,958 per person) and highest in those with CKD stage 5 (AUD$67,117 per person). Inpatient care contributed to the majority (76%) of all healthcare costs. Predictors of increased total annual healthcare cost included more advanced stages of CKD, and the presence of comorbidities. In CKD stage 5, the additional cost per person per year was + $53,634 (95%CI 32,769 to 89,482, *p* < 0.001) compared to people in the at risk group without CKD.

**Conclusion:**

The total healthcare costs in advanced stages of CKD is high, even when patients are not on dialysis. There remains a need for effective primary prevention and early intervention strategies targeting CKD and related chronic conditions.

**Supplementary Information:**

The online version contains supplementary material available at 10.1186/s12913-024-11258-8.

## Background

Chronic kidney disease (CKD) is defined as abnormal kidney structure or function, present for 3 months or more [1]. Early asymptomatic stages of CKD can progress to end-stage kidney disease (ESKD) requiring kidney replacement therapy (KRT) in the form of haemodialysis, peritoneal dialysis, or renal transplantation. Although life sustaining, ESKD requiring KRT has substantial impacts on an individual’s quality of life – this is especially true for Aboriginal and Torres Strait Islander people (hereby respectfully referred to as First Nations people). In the Northern Territory (NT), many First Nations people leave their homes and small remote communities, in order to receive dialysis in urban centres [2–4]. Effective primary health care, screening, and evidence-based management can slow or prevent CKD progression [1].

The NT, where 31% of the population are First Nations Australians, is a hotspot for CKD. CKD disproportionally affects First Nations people. A national Australian Health Survey showed that First Nations people in the NT had a biomedical CKD prevalence of up to 32%, compared to a national average of approximately 10% amongst non-First Nations Australians [5]. A recent NT Health report highlighted similar differences in prevalence between First Nations and non-First Nations adults [6]. The incidence of ESKD is also much higher in First Nations people in the NT, compared to national estimates for First Nations Australians (3.96 versus 0.95 per 1000 population) [7]. In Australia and globally, the disproportionately high burden of CKD in First Nations people is associated with socioeconomic inequities – such as low income, geographic remoteness, limited housing and education, and difficulties in accessing specialised health services [8–11].

The enormous economic burden of KRT in the NT has been well established [12–15]. Gorham et al. showed that on average, a single patient on maintenance dialysis (urban centres) had a total healthcare cost of more than AUD$100,000 per year [15]. Furthermore, the total cost of KRT to the healthcare system has grown rapidly over the past two decades, as demand for dialysis has increased across the NT [16]. Despite what is already known about the costs of KRT, the cost of healthcare for people living with CKD in non-KRT cohorts is less well characterised. A 2020 systematic review of CKD costing studies in developed countries showed that few studies included earlier stages of CKD, and there was great variability in total healthcare cost estimates across different healthcare settings [17]. A majority of included studies were from the USA, and only one was from Australia. In the Australian study, Wyld et al. estimated the annual cost of CKD across all stages of CKD for the AusDiab cohort (6,138 individuals) [18]. Although AusDiab was a national cohort study, there was only a very small proportion of First Nations participants (35 people, 0.6%) within its follow-up cohort [19]. More recently, CKD Queensland (CKD.QLD) published a registry study of hospitalisations and inpatient costs of pre-dialysis patients with CKD, and included 6.6% of First Nations participants [20].

We sought to address this knowledge gap by estimating the healthcare costs in CKD patients without KRT, in the NT context. Estimating cost in CKD across all stages is important for understanding the economic burden of CKD to the health system and the impact of early-intervention strategies, such as improved early detection of CKD across Australia [21]. Establishing costs in patients with earlier stages of CKD in the NT will also provide the baseline costs for future cost-effectiveness studies.

## Methods

### Aims

The aim of this study was to describe the healthcare use and associated costs of people at risk of CKD or living with CKD in the NT, from a healthcare funder perspective.

### Study design

This was a retrospective cohort study of patients within the Territory Kidney Care (TKC) database as of 1 January 2017. The patients were followed up for one year, until 31 December 2017. The TKC database is a live database that is updated daily. Data was extracted from the TKC database on 9 June 2023 for this retrospective cohort.

### Setting and data source

The NT covers a large land mass of approximately 1.4 million km^2^, [22] with a relatively small and dispersed population of 250,000 people [23]. According to the 2021 census, First Nations people represent 31% of the NT population, and 77% of First Nations people in the NT live in remote and very remote areas [23]. Care for people with CKD and related chronic conditions is particularly challenging in remote areas of the NT. Disruptions to care can arise due to a high turnover of staff, high mobility of patients between health services, and electronic health record (EHR) data siloes across services [24, 25]. The TKC project commenced in 2017, aiming to address these challenges and improve care for people with CKD and related chronic conditions. The costs in this study represents a baseline cost in 2017 for patients at risk of CKD and with CKD in the NT, pre-TKC project implementation. Briefly, the TKC project involved establishing a linked EHR database for participating health services in the NT, developing clinical decision support tools using the consolidated EHR data with the TKC database, and partnering with health services to implement these digital tools into clinical practice [26, 27].

The TKC database, conceptually similar to a CKD clinical registry that aims to improve patient care, consolidates EHR data from both government and non-government sources. This includes 1) Caresys – a centralised hospital information system used at all public hospitals (*n* = 6); 2) all publicly funded remote primary health care services (*n* = 56) using the centralised Primary Care Information System (PCIS); and 3) participating Aboriginal Community Controlled Health Services (ACCHS) in the NT (*n* = 11 out of 13 ACCHS) – each using separate implementations of Communicare (Telstra Health) clinical information systems.

For this costing study, individual patient demographics, CKD stages, and co-morbidities were extracted from the TKC database. NT Health provided hospital data for emergency department (ED), inpatient, and hospital outpatient costs. Patients were linked using their Hospital Registration Number (HRN), a unique patient identifier used across the NT, which has been shown to be highly accurate for demographic data in previous validation studies [28].

### Inclusion and exclusion

Our costing study included individuals within the TKC database who had a risk factor for CKD (including acute kidney injury, diabetes, hypertension, and cardiovascular disease) [29], or with CKD stages 1 to 5. Included patients were > 16 years of age, and active in the TKC database as of 1 January 2017. Active was defined as having one or more visits or new EHR data entry within the past 2 years, at a participating health service. Given recent costing studies of KRT patients in the NT [15], we excluded people with CKD who were already on KRT at study baseline.

We used previously published EHR-based algorithms to identify individuals with CKD and at risk of CKD [26]. The development and validation of CKD, KRT, diabetes, hypertension, and cardiovascular disease algorithms are fully outlined in our previous publication. For example, individuals are assigned a CKD stage according to Kidney Disease: Improving Global Outcomes (KDIGO) guidelines using both estimated glomerular filtrate rate (eGFR) for G-staging and urine albumin-to-creatinine ratio (uACR) for A-staging [1]. The CKD algorithm also took into account an individual’s International Classification of Diseases Australian Modified (ICD-10 AM) [30] and primary care International Classification of Primary Care (ICPC-2 PLUS) coded diagnoses for CKD [31]. Individuals with KRT were identified and excluded from the CKD cohort based on administrative and procedural codes for KRT.

### Costing approach

The costing study was conducted from the perspective of the healthcare payer (NT Health, and Australian federal government), and included direct healthcare costs incurred throughout the year. Given that the TKC database primarily extracts structured EHR data, indirect costs such as transportation and out of pocket patient costs, and societal costs (i.e. productivity losses) were not included in the analysis. A bottom-up gross costing approach was used – where individual-level data was used to identify and measure costs, and valuation used a combination of aggregate costs (e.g. for hospitalisations) and unit costs (e.g. for medications). Costs are expressed in $AUD for the year 2023 unless otherwise stated. Where required, we used Australian Institute of Health and Welfare (AIHW) deflators to standardise costs to 2023 dollars. The 2020/21 AIHW deflator was used where more recent deflators were unavailable [32].

For hospital costs, activity-based costing in the form of national weighted average units (NWAUs) was used. The NWAUs associated with each Australian Refined Diagnosis Related Groups (AR-DRG) and urgency related group (URG) were used to cost inpatient and ED episodes, respectively [33]. To avoid double-counting of costs, ED-related URGs was excluded where the ED presentation resulted in an inpatient admission (a DRG cost). NT Health also assigns a NWAU to each hospital outpatient episode, which was used for outpatient costing. NWAUs for inpatients, ED, and outpatients were multiplied by the national efficient price (NEP) for financial year 2022/23 ($5,797).

Primary health care, medication, and laboratory data were extracted from the TKC database. Primary health care-relevant Medical Benefits Schedule (MBS) reimbursement items (see Supplementary Table S1) were multiplied by corresponding MBS unit costs to identify total primary health care costs [34]. Laboratory costs were estimated at $10 per laboratory test [34]. For medication costing, we used unit costs from the Pharmaceutical Benefits Scheme (PBS) Dispensed Price for Maximum Quantity (DPMQ) [35]. Individual medications were matched against PBS reimbursement costs using a combination of string matching (using regular expression algorithms to standardise medication names), and matches based on National Institutes of Health RxNorm RxCUI (Concept Unique Identifier) codes. The RxCUI is a unique identifier assigned to individual drug entities [36]. DPMQ costs were assumed to be a monthly price, with per year costs calculated based on duration of medication use. Erythropoietin was costed separately with an assumption of fortnightly use of darbepoetin alfa, and monthly use of methoxy polyethylene glycol-epoetin beta based on expert opinion. This medication costing strategy was validated against the previous costing study of NT KRT patients, where medications were manually matched to PBS costs [14]. Outpatient radiology costs were available for public hospital-based investigations only, and costed using MBS reimbursement values [34].

### Statistical analysis

Statistical analysis was conducted in Python (v3.9.12; Python Software Foundation) [37] and R (v4.2.3; R Core Team) [38]. Descriptive statistics were used to describe demographics and comorbidities. Mean and standard deviations (SD) were reported for continuous variables, and frequency (percentage) for categorical data. Mean total healthcare costs, and healthcare use per person over the 12-months period was calculated. Statistical significance was set at α = 0.05 throughout.

Due to the high cost of KRT in patients at late stages of CKD, inpatient costs were examined with and without a principal diagnosis of renal dialysis (ICD Z49.1). Subgroup analyses were conducted for those with primary health care-linked data (primarily remote, First Nations individuals) and those without primary health care-linked data (primarily Darwin-based, majority non-First nations individuals).

The association between high total healthcare cost and demographics, comorbidities, and CKD stages was explored using several statistical models. Six potential models were fitted – 3 were generalised linear models (GLMs), and 3 were two-part GLMs. The two-part GLMs are common in costing studies, where zero costs and non-zero costs are modelled separately [39]. Based on several model accuracy parameters, the final model selected was a GLM with an identity link and gamma distribution. See Supplementary Table S2 and Supplementary Figure 1 for model goodness of fits. Sensitivity analysis on the cost prediction model was conducted by fitting the final model on data from those with and without primary health care-linked data.

### Ethics

The study protocol for the TKC project evaluation, including economic evaluation, was approved by the Human Research Ethics Committee of NT Health and Menzies School of Health Research (NTHREC 2021–4102).

## Results

### Overview

**Fig. 1:**
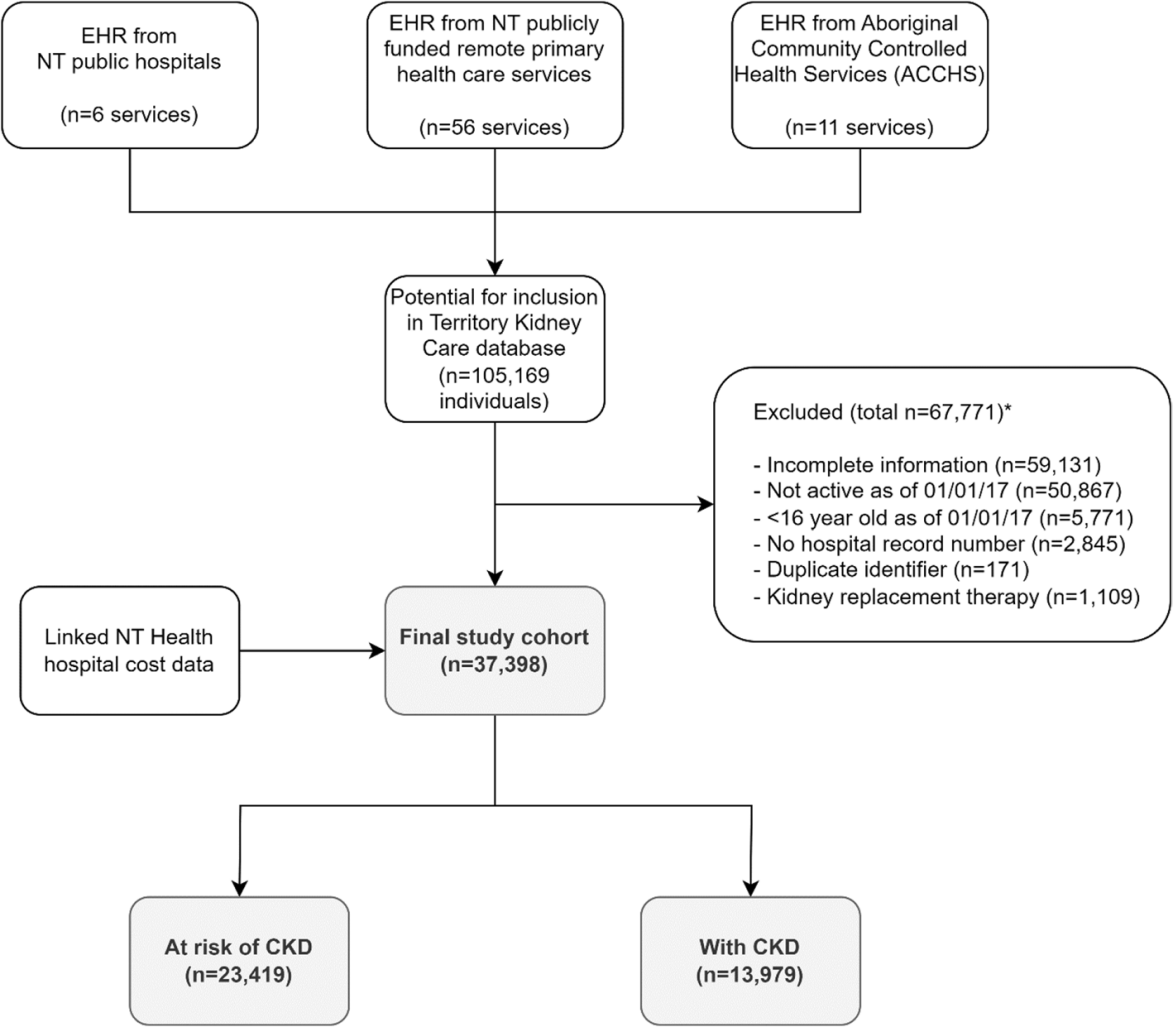
Flowchart of study cohort. *Note that exclusion criteria are not mutually exclusive – i.e. patients may have had one or more reasons for exclusion. Abbreviations: CKD – chronic kidney disease; EHR – electronic health record; NT – Northern Territory

Of the patients considered for inclusion in the TKC database (*n* = 105,169), 37,398 individuals met the study criteria (Fig. 1). Common reasons for exclusion included insufficient information to be assessed for inclusion (e.g. single decreased eGFR reading), and being inactive (defined as did not attend or have an EHR data entry at an included health service in the 2 years prior to 1 January 2017). Of the included cohort, 23,419 (63%) were at risk of CKD, and 13,979 (37%) had CKD (stages 1 to 5). Of those at risk of CKD, common co-morbidities included diabetes (36%), hypertension (32%), and coronary artery disease (11%). Of those with CKD, there were more participants in earlier compared to late stages of CKD (Table 1). The overall mean (± SD) age of participants in this study was 45 years (± 17). There were similar proportions of males and females (49% males), and a large proportion of First Nations people were included (68%). Primary health care (PHC) data was available for 62% of the overall cohort.

**Table 1:** Baseline characteristics of study cohort at 1 January 2017

	**At risk**	**CKD 1**	**CKD 2**	**CKD 3a**	**CKD 3b**	**CKD 4**	**CKD 5**	**Overall**
Number of people (n)	23,419	7,445	3,560	1,642	752	394	186	37,398
Percentage of cohort (%)	63%	20%	9%	4%	2%	1%	< 1%	100%
**Demographics – mean (SD) or proportion (%)**								
Age – mean (SD)	45 (17)	36 (12)	54 (13)	65 (14)	66 (14)	62 (15)	58 (14)	45 (17)
Male	53%	36%	52%	50%	48%	44%	39%	49%
First Nations	61%	91%	80%	47%	53%	62%	76%	68%
PHC-linked data	54%	84%	82%	42%	42%	46%	48%	62%
**Demographics – prevalence (%)**								
Diabetes	30%	37%	53%	53%	62%	70%	73%	36%
Hypertension	25%	29%	58%	59%	69%	77%	77%	32%
Coronary artery disease	9%	6%	19%	24%	26%	33%	31%	11%
Cerebral vascular disease	1%	2%	4%	3%	3%	5%	2%	2%
Peripheral vascular disease	1%	1%	2%	4%	5%	6%	5%	2%
Obesity (BMI > 30 kg/m^2^)	19%	31%	25%	12%	12%	14%	17%	21%
Rheumatic heart disease	6%	9%	8%	7%	9%	9%	9%	7%
Multimorbidity^a^	23%	35%	55%	52%	61%	71%	73%	31%

*Abbreviations*: *BMI* Body mass index, *CKD* Chronic kidney disease, *PHC* Primary health care, *SD* Standard deviation

^a^Multimorbidity is defined as >  = 2 related conditions (diabetes, hypertension, coronary artery disease, cerebral vascular disease, peripheral vascular disease, obesity, rheumatic heart disease). Rounded to whole numbers

The proportion of people with related chronic conditions (e.g. diabetes, hypertension, coronary artery disease) tended to increase with the severity of CKD. For example, a high number of people in CKD stage 5 had comorbid hypertension (77%), diabetes (73%), and coronary artery disease (31%). The proportion of people with multimorbidity increased across the CKD stages – with most people in CKD stage 5 (73%) having at least two related chronic conditions.

### Healthcare use

As expected, average healthcare use over the 12 months period increased progressively with CKD stage (Table 2). The total number of inpatient admissions rose from 0.8 inpatient admissions for patients at risk of CKD, up to 28.2 in patients with CKD stage 5 (3.6 when excluding dialysis admissions). Most other health service utilisation categories (e.g. ED, outpatients, medications, labs) also increased with a progression in CKD stage. Primary health care visits and outpatient radiology did not increase with CKD stage, and this may have been due to missing primary health care-linked data in in the overall cohort. However, within the subgroup with primary health care-linked data, there was a clear increase in primary health care visits with a progression in CKD stage (Supplemental Table S8).

**Table 2:** Average annual healthcare use per person (mean, SD)

**Mean visits (SD)**	**At risk**, *n* = 23,419	**CKD 1**, *n* = 7,445	**CKD 2**, *n* = 3,560	**CKD 3a**, *n* = 1,642	**CKD 3b**, *n* = 752	**CKD 4**, *n* = 394	**CKD 5**, *n* = 186	**Overall**, *n* = 37,398
ED	0.6 (1.5)	0.7 (1.8)	0.8 (2.0)	0.8 (1.9)	1.1 (2.3)	1.6 (2.5)	3.5 (7.4)	0.7 (1.8)
Inpatient	0.8 (2.2)	0.8 (1.9)	1.0 (3.5)	1.2 (3.4)	1.5 (3.9)	5.1 (13.4)	28.2 (43.5)	1.0 (4.6)
→ Dialysis only	0.0 (0.9)	0.0 (0.2)	0.1 (2.6)	0.1 (2.4)	0.1 (2.7)	3.2 (12.1)	24.7 (41.0)	0.2 (3.8)
→ Inpatient (no dialysis)	0.8 (2.0)	0.8 (1.9)	1.0 (2.2)	1.2 (2.3)	1.3 (2.7)	1.8 (2.7)	3.6 (6.8)	0.8 (2.1)
Outpatient	2.0 (5.8)	1.2 (4.0)	2.1 (5.0)	3.4 (7.7)	3.5 (5.8)	5.6 (8.3)	8.0 (16.3)	2.0 (5.7)
Primary care	1.2 (1.9)	2.4 (2.5)	3.1 (2.9)	1.7 (2.7)	2.0 (3.0)	2.1 (3.1)	2.0 (2.9)	1.7 (2.3)
Medications	2.7 (4.1)	5.1 (5.2)	5.7 (6.1)	3.4 (5.8)	4.3 (6.8)	6.6 (9.2)	10.1 (11.2)	3.6 (5.0)
Labs (individual tests)	20.3 (33.9)	32.2 (38.5)	41.0 (47.1)	37.9 (59.8)	50.0 (65.0)	72.9 (85.8)	102.6 (116.1)	27.0 (41.6)
Radiology	0.1 (1.0)	0.1 (1.1)	0.1 (1.0)	0.1 (0.9)	0.0 (0.4)	0.1 (0.7)	0.2 (1.4)	0.1 (1.0)

Radiology is outpatient radiology only. Numbers are rounded to 1 decimal place. Continuous variables expressed in mean (SD)

*Abbreviations*: *CKD* Chronic kidney disease, *ED* Emergency department

### Healthcare costs

**Fig. 2:**
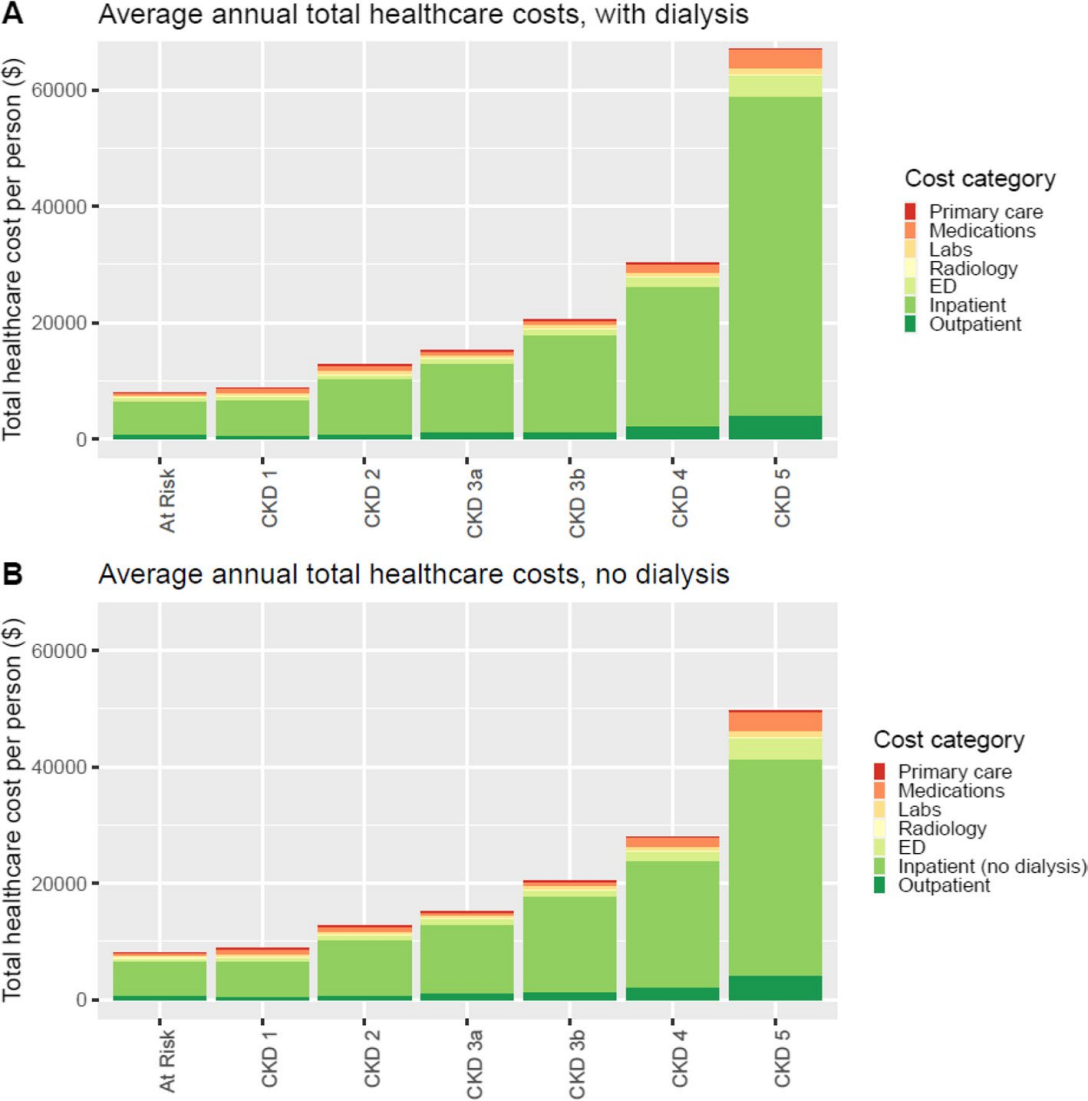
Average annual total healthcare costs per person, by CKD stage

Similarly, mean healthcare costs increased with CKD stage, with highest average annual total healthcare costs in CKD stage 5 ($67,117 including dialysis admissions, $49,614 excluding dialysis admissions) – Fig. 2A and B. There was a sharp increase in total healthcare costs from CKD stage 4 to CKD stage 5. Inpatient costs contributed to most total healthcare costs (77%) (Table 3). About a third of patients with CKD stage 5 not on KRT at entry of the study (37%) started dialysis throughout the 1-year follow-up period. In these patients, the inpatient costs of dialysis (ICD Z49.1) was estimated as $17,503 per person per year. For medication costs, erythropoietin was a high-cost item, accounting for approximately one-third of total medication costs in CKD stage 5. The sum of total annual healthcare costs was $362 million for the study cohort, with $186 million for the at risk group, and $176 million for the CKD cohort (Supplementary Table 3).

**Table 3:** Average annual total healthcare costs per person (mean, SD)

**Mean cost (SD)**	**At risk**, *n* = 23,419	**CKD 1**, *n* = 7,445	**CKD 2**, *n* = 3,560	**CKD 3a**, *n* = 1,642	**CKD 3b**, *n* = 752	**CKD 4**, *n* = 394	**CKD 5**, *n* = 186	**Overall**, *n* = 37,398	**Overall**, % of total costs
ED	542 (1,532)	625 (1,579)	783 (1,911)	896 (1,868)	1,139 (2,284)	1,602 (2,629)	3,615 (7,693)	636 (1,734)	7%
Inpatient	6,040 (23,092)	6,340 (20,914)	9,701 (30,386)	12,062 (35,584)	16,698 (46,886)	24,154 (47,719)	54,988 (79,258)	7,361 (26,122)	76%
→ Dialysis only	6 (605)	1 (129)	49 (1,910)	49 (1,573)	103 (1,923)	2,330 (8,752)	17,503 (29,119)	124 (2,707)	1%
→ Inpatient (no dialysis)	6,034 (23,075)	6,339 (20,914)	9,651 (30,220)	12,012 (35,518)	16,594 (46,660)	21,824 (44,183)	37,484 (67,170)	7,237 (25,700)	75%
Outpatient	530 (1,685)	340 (1,199)	583 (1,683)	940 (2,081)	1,129 (2,054)	2,017 (3,900)	3,961 (11,739)	560 (1,885)	6%
Primary care	167 (352)	333 (447)	450 (584)	261 (526)	301 (568)	313 (560)	289 (495)	236 (427)	2%
Medications	459 (1,302)	828 (1,336)	857 (1,464)	596 (2,320)	745 (1,508)	1,465 (2,589)	3,215 (4,522)	607 (1,465)	6%
Labs	203 (339)	322 (385)	410 (471)	379 (598)	500 (650)	729 (858)	1,026 (1,161)	270 (416)	3%
Radiology	16 (167)	16 (162)	16 (169)	15 (147)	5 (85)	5 (39)	24 (233)	16 (164)	0%
Total	7,958 (24,729)	8,804 (22,684)	12,802 (32,687)	15,149 (37,813)	20,516 (48,949)	30,285 (52,168)	67,117 (91,367)	9,685 (28,201)	100%

Radiology is outpatient radiology only. $AUD 2023 dollars, rounded to whole dollars. Percentages are rounded to whole numbers. Continuous variables expressed in mean (SD)

*Abbreviations*: *CKD* Chronic kidney disease, *ED* Emergency department

### Cost prediction model

A multivariable GLM was used to predict total annual healthcare costs (Table 4). The final regression model included age; sex; First Nations status; presence of diabetes, hypertension, cardiovascular disease (including coronary artery disease, cerebral vascular disease, peripheral vascular disease); and CKD stage. Even when adjusted for demographic and co-morbidity variables, more advanced stages of CKD were important predictors of total healthcare costs. Notably, in the cost model an individual with CKD stage 5 had, on average, an estimated cost of + $53,634 (32,769 – 89,482) per year more in additional total healthcare costs, compared to a person at risk of CKD but without CKD (*p* < 0.001). Co-morbidities were also significant predictors of cost, with the highest additional costs incurred by those with cardiovascular disease (+ $5,827), followed by those with diabetes (+ $2,423 per year), and hypertension (+ $1,451 per year).

**Table 4:** Cost prediction model – multivariable generalised linear model (identity link, gamma distribution)

Variable	Additional cost per person per year, adjusted (95% CI)	*P*-value
Age (for each additional year)	+ $104 (92 – 115)	< 0.001
Male	+ $310 (-43 – 670)	0.14
First Nations	+ $3,600 (3,234 – 3,973)	< 0.001
Diabetes	+ $2,423 (1,824 – 3,058)	< 0.001
Hypertension	+ $1,451 (783 – 2,172)	< 0.001
Cardiovascular disease	+ $5,988 (4,625 – 7,487)	< 0.001
CKD stage 1^a^	+ $250 (-184 – 737)	0.31
CKD stage 2	+ $1,034 (91 – 2,121)	0.03
CKD stage 3a	+ $2,286 (692 – 4,237)	< 0.01
CKD stage 3b	+ $5,827 (2,634 – 10,066)	< 0.001
CKD stage 4	+ $15,953 (9,261 – 25,726)	< 0.001
CKD stage 5	+ $53,634 (32,769 – 89,482)	< 0.001

*Abbreviation*: *CKD* Chronic kidney disease

^a^Comparison group for all CKD stages is people at risk of CKD. Cardiovascular disease refers to any individual with coronary artery disease, cerebral vascular disease, and/or peripheral vascular disease. Costs are in $AUD 2023 dollars, rounded to whole dollars

### Subgroup analysis – with and without primary health care-linked data

Of the overall study cohort, 23,195 (62%) individuals had primary health care-linked data. The primary health care-linked data were mostly from remote health services outside of the Darwin region, which were either government-funded primary health clinics, or ACCHS primary health clinics. Mean age was younger in the subgroup with primary health care-linked data (42 years) compared to those without primary health care data (51 years). There were proportionally more First Nations people in those with primary health care data (89%) – nevertheless, there was still a-third of patients who identified as First Nations in the group without primary health care-linked data (36%). Proportions of people with co-morbidities was higher in those with primary health care-linked data (35% versus 25% respectively, with two or more related chronic conditions) – possibly reflecting a more complete profile of coded diagnoses within the TKC database, or reflecting a true increase in prevalence of chronic diseases within the remote First Nations populations in the NT. See Supplementary Tables and Figures for full results of the subgroup analysis.

Overall, average annual costs were lower in those with primary health care-linked data ($6,417 per person), compared to those without primary health care-linked data ($9,801 per person). However, this pattern was reversed in late CKD stages with higher costs in the subgroup with primary health care-linked data. Our hypothesis for this observed pattern is that people from remote areas are likely to be underserved in earlier stages of CKD but have higher healthcare use in later stages. For CKD stage 5 there was a doubling of total healthcare costs between those with ($96,632) and those without primary healthcare-linked data ($40,037). This reflects our clinical experience in the NT, where people with ESKD from remote areas are more likely to progress rapidly to dialysis and commence KRT in an unplanned fashion – resulting in higher inpatient costs and poorer patient outcomes [40].

## Discussion

### Main findings and implications

The total annual healthcare cost within our cohort of CKD patients was $176 million. We found that average annual healthcare costs increased with CKD disease progression, and inpatient costs accounted for the majority of healthcare costs. Even when inpatient dialysis costs are excluded, the total annual healthcare cost in ESKD is high ($49,614 per person for CKD stage 5). High costs in ESKD likely reflect the heavy burden of multimorbidity and associated hospitalisations in this population. In CKD stage 5, total annual healthcare costs per person are close to double for remote First Nations people, likely reflecting the challenges in integration of care to provide an optimal patient journey, especially for patients transitioning to commence KRT. Although the costs of advanced stages of CKD are striking, the economic burden of patients in the at risk and earlier CKD cohorts should not be overlooked. The at risk group had the lowest average annual costs in the NT, but still had an inpatient cost ($7,958 per person) that was substantially higher than the AIHW national estimates for inpatient costs ($3,497 per person, 2020–21) [32].

Our results have resource allocation implications. Along with what is already known about the high cost of dialysis patients in the NT (over $60 million total annual healthcare costs for the 2017 KRT cohort) [15] our results strengthen the argument to invest resources to address social determinants of health, primary prevention, early identification of CKD, and slowing CKD progression through optimal management of risk factors and associated chronic conditions. The implementation arm of the TKC project seeks to improve these aspects of CKD care through strengthening partnerships across primary and tertiary health services in the NT, improved EHR data sharing (TKC database), and use of digital innovation such as individual and service-level clinical decision support tools [26, 27].

### Comparisons with previous studies

In our cohort, average annual healthcare costs ranged from $7,958 in people at risk of CKD to $67,117 in patients with CKD stage 5. In CKD stage 5, the non-dialysis costs (approximately $50,000) is almost the same as non-dialysis inpatient costs incurred by KRT patients in the NT [15]. A recently published Queensland CKD registry study of pre-dialysis patients with CKD showed similar increases in inpatient costs with CKD progression. However, our inpatient costs across each CKD stage were approximately 1.5 times higher than that of the Queensland registry cohort [20]. Our estimate of average annual healthcare costs per patient are also higher than the 2004/2005 AusDiab cohort study, which estimated direct total healthcare costs to range from $1,829 per person ($2,260 in 2023) for those without CKD to $14,545 per person ($17,975 in 2023) in late stages of CKD (stages 4, 5) [18]. As previously highlighted, our cohort differed from the AusDiab cohort, which had very few First Nations and remote NT participants [18].

In a Kidney Health Australia commissioned report, annual 2021 healthcare costs of people with CKD stages was estimated to range from $41 per person in early stages (CKD stages 1 and 2) to $62,358 per person in CKD stage 5 [21]. The costing methodology used contributed to different estimates of costs. For example, we directly costed all healthcare costs (both CKD and non-CKD related) whereas the Kidney Health Australia report included inpatient admissions related to CKD only by attributing a proportion of hospitalisations to CKD amongst all hospitalisations (from 0% in early stages, to 50% in late stages). Elshahat et al. demonstrated that internationally, CKD progression was associated with higher total healthcare costs (up to 1.3–4.2-fold for CKD stages 4 and 5). However, the systematic review reported that actual annual healthcare cost per person varied greatly depending on the population studied, the country, and healthcare setting [17].

### Strengths and limitations

Few studies to date have examined the cost of CKD across at risk populations, as well as all stages of CKD. We analysed a large comprehensive dataset of patients in the NT with a risk factor for CKD, or with CKD, and included individual-level linked healthcare costs from both hospitals and primary health care. Importantly, given the large proportion of First Nations Australians within our cohort, this study provides a first comprehensive insight into healthcare costs for First Nations Australians living with CKD in the NT.

However, there are limitations with using routinely collected EHR data for secondary purposes. Firstly, not all ACCHS or private GP practices in the NT are currently participating in the TKC project. Private specialist outpatients and private hospital data are also not captured within the TKC database. Although we have a large proportion of the NT adult population included in this costing study, our results do not reflect the entire NT population. Healthcare use, such as visits to non-participating health services, are not captured in our costing study. The TKC project continues to partner with additional ACCHS and private GP practices across the NT. These collaborations are key to improving data comprehensiveness and representativeness, and will enable future work in estimating whole-of-NT CKD prevalence and costs.

Secondly, use of routinely-collected EHR data is subject to data quality issues – including issues of data completeness, correctness, concordance, plausibility, and currency [41, 42]. For example, where an individual has sparse EHR data, it is not possible to determine whether healthcare use data was missing (e.g. moved and used health services interstate), or if the patient simply did not require healthcare services throughout the follow-up period. To improve data quality, we restricted the costing cohort to include participants who had an active EHR entry within the past 2 years (prior to 1 January 2017). TKC project staff are also allocated to check, validate, and conduct continuous quality improvement of the data within the TKC database. In terms of data currency, our data available for analysis was a limited 12-month window in 2017 – future studies could use more contemporary data, or data over a longer period of time to improve generalisability of the costing results.

Thirdly, most but not all MBS items commonly used in primary care were available for analysis in our study. This contributed to an underestimation of total primary health care costs. More importantly, using MBS reimbursement costs to estimate cost of primary health care services, is problematic in remote NT settings. Zhao et al. estimated that for a First Nations person living in remote areas of the NT, there is a Medicare shortfall of $531 to $1,041 per annum compared with a non-First Nations person living in an urban area [43]. This is due to the model of care in many remote NT communities, where doctors are only available for some sessions of the week, and care is primarily Aboriginal health practitioner (AHP) and nurse-led. Thus, MBS fee-for-service estimates, where many MBS items can only be claimed by GPs, underestimates the actual costs of care provision in these settings. To better estimate actual costs of delivering primary health care, the NT Health are currently updating a 2006 costing study of remote primary health care services in the NT [44].

Fourthly, we compared the total healthcare costs of patients at risk of CKD, and across CKD stages, but our costing methodology does not specify which costs were directly attributable to CKD. Future work can focus on detailing high cost and high use items across each category (e.g. DRGs, PBS medications). In the Queensland CKD registry study, dominant contributors to inpatient costs included cardiovascular and CKD-related conditions [20]. CKD exists in the context of multimorbidity, and is both the “villain and victim” of related comorbidities such as hypertension, and cardiovascular conditions [45, 46]. Thus, additional assumptions would need to be introduced to apportion healthcare use and costs related specifically to CKD, versus costs related to other closely related chronic conditions.

Finally, direct healthcare costs are only part of the picture, in terms of the economic impact of CKD. Furthermore, our costs do not capture the significant burden of CKD to patients and their communities – including financial impacts of travel time, relocation, carer needs, and productivity losses [2–4]. Indirect healthcare costs, such as publicly funded patient travel, are not included. Patient travel borne by NT health includes eligible travel and accommodation from remote communities to regional hospitals, and travel from NT hospitals to interstate hospitals for specialised care. Patient travel costs can account for up to one-fourth of the costs per admission, across several hospitals in the NT. Thus, the true cost of delivering healthcare for individuals is likely to be substantially higher if the costing perspective considered patient travel costs.

## Conclusions

Our study showed that annual healthcare costs ranged from $7,958 per person in people at risk of CKD, to $67,117 per person in people with CKD stage 5. Combining our conservative estimates of health system expenditure on the 2017 pre-dialysis cohort (CKD Stages 1 to 5), with that for the 1,000 people currently receiving KRT for ESKD, in 2023 the annual cost of providing health care for people living with CKD in the NT is more than $250 million. There is a strong imperative for primary prevention, improved screening, and optimal management strategies in early CKD. Such strategies could benefit patients and reduce the financial burden on healthcare systems.

### Supplementary Information

Supplementary Material 1.

Supplementary Material 2.

## Data Availability

Data is not publicly available due to privacy considerations. Permission for datasets used in this study can be sought from data custodians including NT Health (https://health.nt.gov.au/data-and-research/nt-health-research/data-access), Territory Kidney Care (https://www.menzies.edu.au/page/Research/Projects/Kidney/territory_kidney_care/), and individual health services.

## Abbreviations

ACCHS: Aboriginal Community Controlled Health Services
AHP: Aboriginal health practitioner
AIHW: Australian Institute of Health and Welfare
AUD: Australian dollars
CKD: Chronic kidney disease
CKD-EPI: Chronic Kidney Disease Epidemiology Collaboration
DPMQ: Dispensed Price for Maximum Quantity (on PBS)
eGFR: Estimated glomerular filtration rate
ED: Emergency department
EHR: Electronic health record
ESKD: End-stage kidney disease
GLM: Generalised linear models
GP: General practice
ICD: International Classification of Disease
ICPC: International Classification of Primary Care
KDIGO: Kidney Disease Improving Global Outcomes
MBS: Medicare Benefits Schedule
NEP: National efficient price
NWAU: National weighted average units
NT: Northern Territory
PHC: Primary health care
PBS: Pharmaceutical Benefits Scheme
KRT: Kidney replacement therapy
TKC: Territory Kidney Care
uACR: Urine albumin-to-creatinine ratio
URG: Urgency related group
