# Supplementary material for: Costs and healthcare use of patients with chronic kidney disease in the Northern Territory, Australia
Source: BMC Health Serv Res. 2024 Jul 9;24:791. doi: 10.1186/s12913-024-11258-8 (PMC11234693; doi:10.1186/s12913-024-11258-8)
Supplement: Supplementary file 1 — Supplementary Material 1. [file 12913_2024_11258_MOESM1_ESM.pdf]

Additional files 1 – tables and figures

Manuscript: Costs and healthcare use of patients with chronic kidney disease in the Northern Territory, Australia

Authors: Winnie Chen, Kirsten Howard, Gillian Gorham, Asanga Abeyaratne, Yuejen Zhao, Oyelola Adegboye, Nadarajah Kangaharan, Mohammad Radwanur Rahman Talukder, Sean Taylor, Alan Cass, Territory Kidney Care Steering Committee

Table of contents:

Table S1: List of included Medicare Benefits Scheme item numbers

Table S2: Cost prediction model goodness of fits – model diagnostics

Figure S1: Cost prediction model goodness of fits – residual plots

Table S3: Sum of total annual healthcare costs in the study cohort, by CKD stage

Table S4: Baseline characteristics of study cohort at 1 January 2017 – PHC-linked

Table S5: Baseline characteristics of study cohort at 1 January 2017 – no PHC-linked

Table S6: Average annual healthcare use per person (mean, SD) – PHC-linked

Table S7: Average annual healthcare use per person (mean, SD) – no PHC-linked

Figure S2: Average annual total healthcare costs per person, by CKD stage – PHC-linked

Figure S3: Average annual total healthcare costs per person, by CKD stage – no PHC-linked

Table S8: Average annual total healthcare costs per person (mean, SD) – PHC-linked

Table S9: Average annual total healthcare costs per person (mean, SD) – no PHC-linked

Table S1: List of included Medicare Benefits Scheme item numbers

Type	Item number
MBS item	23
MBS item	36
MBS item	44
MBS item	715
MBS item	721
MBS item	723
MBS item	729
MBS item	731
MBS item	732
MBS item	735
MBS item	739
MBS item	743
MBS item	747
MBS item	750
MBS item	758
MBS item	820
MBS item	822
MBS item	823
MBS item	825
MBS item	826
MBS item	828
MBS item	830
MBS item	832
MBS item	834
MBS item	900
MBS item	903

MBS item	10950
MBS item	10951
MBS item	10952
MBS item	10953
MBS item	10954
MBS item	10956
MBS item	10958
MBS item	10960
MBS item	10962
MBS item	10968
MBS item	10983
MBS item	10984
MBS item	10987
MBS item	10991
MBS item	10997

List of explanations for MBS items are available on the MBS Online website.(34) Abbreviations: MBS – Medicare Benefits Scheme

Table S2: Cost prediction model goodness of fits – model diagnostics

Name	Model type	AIC	RMSE	MAE
glm1	GLM identity link, Gaussian distribution	874048	27330	11879
glm2	GLM log link, negative binomial distribution	655718	29759	9671
**glm3**	**GLM identity link, gamma distribution**	**482448**	**27347**	**11880**
tpm1	Overall		27294	11932
	Zeroes - logit	31090		
	Positive values - GLM log link, Gaussian distribution	726444		
tpm2	Overall		27516	11988
	Zeroes - logit	31090		
	Positive values - GLM log link, gamma distribution	726400		
tpm3	Overall		27373	11892
	Zeroes - logit	31151		
	Positive values - GLM log link, Poisson distribution	819943548		
tpm4	Overall		27276	11874
	Zeroes - logit	31151		
	Positive values - GLM identity link, Gaussian distribution	726009		

Bold represents final model selected. Abbreviations: AIC – Akaike Information Criterion; GLM – generalised linear model; MAE – Mean Absolute Error; RMSE – Root Mean Square Error; tpm – two part model.

Figure S1: Cost prediction model goodness of fits – predicted versus residual plots

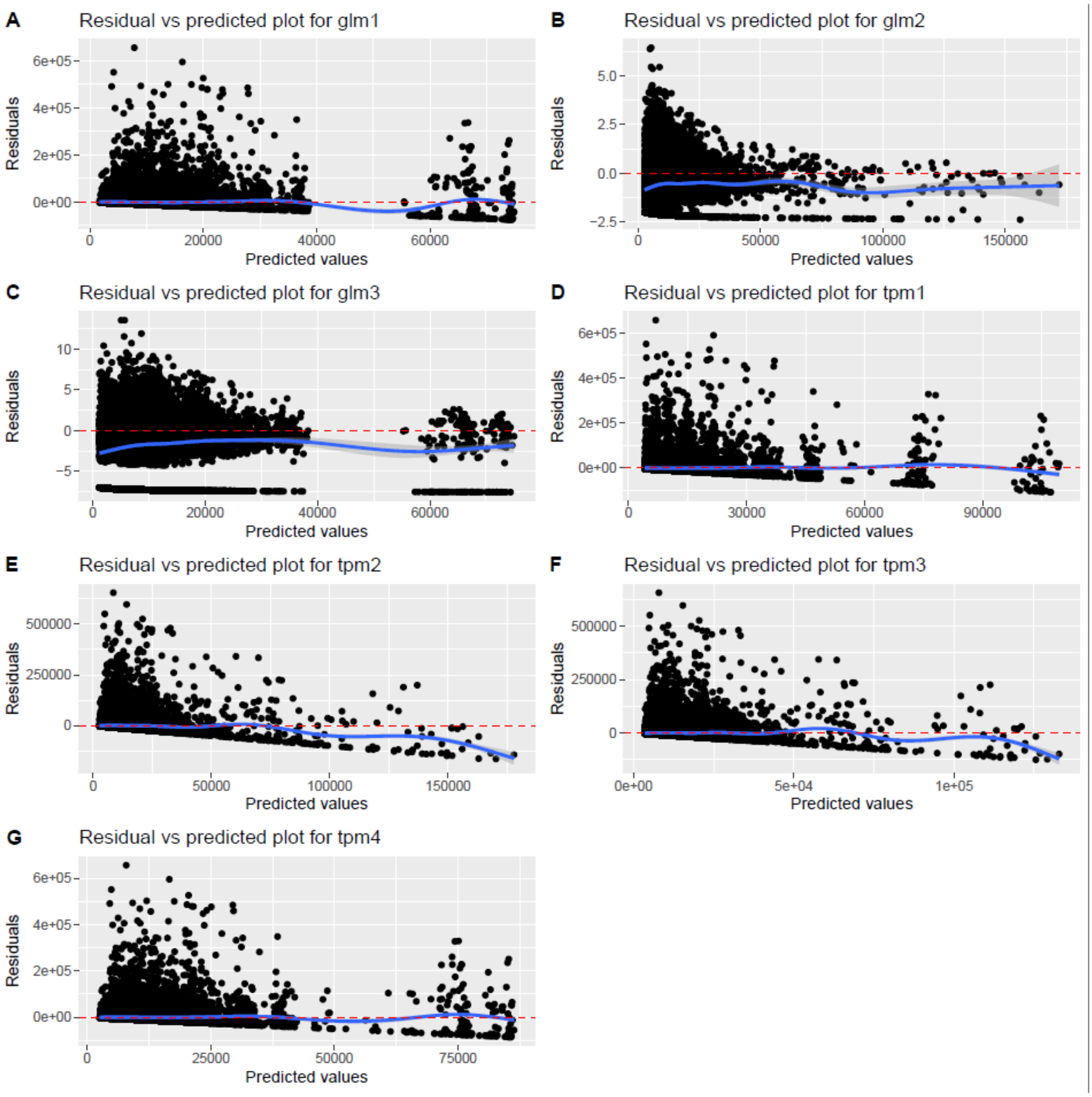

Blue line represents smoothed line of best fit using generalised additive model (GAM) method. Names of models: glm1 – GLM identity link, Gaussian distribution; glm2 – GLM log link, negative binomial distribution; glm3 – GLM identity link, gamma distribution; tpm1 – part 1 logit, part 2 GLM log link, Gaussian distribution; tpm2 – part 1 logit; part 2 GLM log link, gamma distribution; tpm3 – part 1 logit, part2 GLM log link, Poisson distribution; tpm4 – part 1 logit, part 2 GLM identity link, Gaussian distribution.

Table S3: Sum of total annual healthcare costs in the study cohort, by CKD stage ($)

**Total cost ($)**	**At risk**, n = 23,419	**CKD 1**, n = 7,445	**CKD 2**, n = 3,560	**CKD 3a**, n = 1,642	**CKD 3b**, n = 752	**CKD 4**, n = 394	**CKD 5**, n = 186	**Any CKD stage,** n = 13,979	**Overall**, n = 37,398
ED	12,698,517	4,655,689	2,788,867	1,471,807	856,529	631,303	672,435	11,076,630	23,775,150
Inpatient	141,445,842	47,204,201	34,534,957	19,805,209	12,556,859	9,516,540	10,227,684	133,845,450	275,291,294
→ Dialysis only	141,313,877	47,193,062	34,359,203	19,724,192	12,479,053	8,598,477	6,972,109	129,326,096	270,639,976
→ Inpatient (no dialysis)	131,965	11,138	175,753	81,017	77,805	918,062	3,255,574	4,519,349	4,651,318
Outpatient	12,418,565	2,528,013	2,077,065	1,544,030	849,318	794,710	736,682	8,529,818	20,948,387
Primary care	3,918,909	2,478,388	1,603,192	427,908	225,997	123,385	53,708	4,912,578	8,831,490
Medications	10,760,097	6,162,076	3,052,478	979,101	559,895	577,283	597,927	11,928,760	22,688,861
Labs	4,755,230	2,399,680	1,459,730	621,600	375,790	287,050	190,900	5,334,750	10,089,980
Radiology	379,495	119,286	58,636	25,040	3,969	1,935	4,484	213,350	592,847
Total	186,376,657	65,547,336	45,574,928	24,874,698	15,428,359	11,932,208	12,483,823	175,841,352	362,218,012

Radiology is outpatient radiology only. $AUD 2023 dollars, rounded to whole dollars. Abbreviations: CKD – chronic kidney disease, ED – emergency department.

Table S4: Baseline characteristics of study cohort at 1 January 2017 – PHC-linked

	At risk	CKD 1	CKD 2	CKD 3a	CKD 3b	CKD 4	CKD 5	Over all
Number of people (n)	12,749	6,265	2,902	689	318	183	89	23,195
Percentage of cohort (%)	55%	27%	12%	3%	1%	1%	1%	100%
**Demographics – mean (SD) or proportion (%)**								
Age – mean (SD)	40 (15)	37 (11)	53 (13)	61 (14)	61 (14)	58 (14)	55 (12)	42 (15)
Male	48%	37%	50%	42%	37%	37%	31%	45%
First Nations	83%	98%	93%	87%	89%	93%	98%	89%
PHC-linked data	100%	100%	100%	100%	100%	100%	100%	100%
**Demographics – prevalence (%)**								
Diabetes	21%	40%	58%	66%	75%	79%	87%	34%
Hypertension	23%	33%	63%	76%	84%	90%	92%	34%
Coronary artery disease	7%	6%	20%	27%	26%	34%	31%	9%
Cerebral vascular disease	2%	2%	5%	7%	6%	10%	3%	3%
Peripheral vascular disease	1%	1%	2%	3%	4%	2%	4%	1%
Obesity	29%	36%	30%	26%	27%	30%	31%	31%
Rheumatic heart disease	8%	10%	9%	11%	10%	10%	9%	9%
Multimorbidity*	23%	39%	61%	72%	80%	85%	90%	35%

*Multimorbidity is defined as >=2 related conditions (diabetes, hypertension, coronary artery disease, cerebral vascular disease, peripheral vascular disease, obesity, rheumatic heart disease). Rounded to whole numbers. Abbreviations: BMI – body mass index; CKD – chronic kidney disease; PHC – primary health care; SD – standard deviation.

Table S5: Baseline characteristics of study cohort at 1 January 2017 – no PHC-linked

	At risk	CKD 1	CKD 2	CKD 3a	CKD 3b	CKD 4	CKD 5	Over all
Number of people (n)	10,670	1,180	658	953	434	211	97	14,203
Percentage of cohort (%)	75%	8%	5%	7%	3%	1%	1%	100%
**Demographics – mean (SD) or proportion (%)**								
Age – mean (SD)	50 (18)	34 (12)	58 (15)	69 (14)	70 (13)	66 (15)	61 (15)	51 (19)
Male	58%	35%	62%	55%	56%	49%	45%	56%
First Nations	35%	58%	27%	19%	27%	36%	55%	36%
PHC-linked data	0%	0%	0%	0%	0%	0%	0%	0%
**Demographics – n (%Y)**								
Diabetes	41%	17%	32%	44%	53%	61%	61%	40%
Hypertension	26%	12%	35%	47%	58%	66%	63%	28%
Coronary artery disease	12%	2%	17%	23%	26%	33%	30%	13%
Cerebral vascular disease	1%	0%	1%	0%	0%	0%	1%	0%
Peripheral vascular disease	2%	1%	3%	4%	6%	10%	5%	2%
Obesity	6%	8%	3%	1%	1%	1%	4%	6%
Rheumatic heart disease	3%	4%	3%	5%	8%	8%	9%	4%
Multimorbidity*	22%	11%	28%	38%	48%	59%	58%	24%

*Multimorbidity is defined as >=2 related conditions (diabetes, hypertension, coronary artery disease, cerebral vascular disease, peripheral vascular disease, obesity, rheumatic heart disease). Rounded to whole numbers. Abbreviations: BMI – body mass index; CKD – chronic kidney disease; PHC – primary health care; SD – standard deviation.

Table S6: Average annual healthcare use per person (mean, SD) – PHC-linked

**Mean visits (SD)**	**At risk**, n = 12,749	**CKD 1**, n = 6,265	**CKD 2**, n = 2,902	**CKD 3a**, N = 689	**CKD 3b**, n = 318	**CKD 4**, n = 183	**CKD 5**, n = 89	**Overall**, n = 23,195
ED	0.4 (1.4)	0.7 (1.8)	0.8 (1.8)	0.9 (2.1)	1.0 (2.0)	1.9 (3.0)	5.5 (9.4)	0.6 (1.7)
Inpatient	0.6 (1.7)	0.8 (1.8)	1.0 (3.6)	1.1 (2.5)	1.4 (3.9)	6.4 (14.6)	40.6 (45.3)	0.9 (4.5)
→ Inpatient (dialysis only)	0.0 (0.5)	0.0 (0.0)	0.1 (2.9)	0.0 (0.8)	0.2 (3.2)	4.2 (13.2)	35.4 (43.1)	0.2 (3.8)
→ Inpatient (no dialysis)	0.6 (1.6)	0.8 (1.8)	0.9 (2.0)	1.0 (2.3)	1.2 (2.1)	2.2 (3.0)	5.2 (8.3)	0.7 (1.9)
Outpatient	1.1 (3.1)	1.1 (3.8)	1.8 (4.4)	2.3 (5.3)	3.1 (4.4)	5.0 (6.2)	8.4 (12.9)	1.3 (3.8)
Primary care	2.1 (2.2)	2.8 (2.5)	3.7 (2.7)	4.1 (2.9)	4.6 (3.1)	4.4 (3.2)	4.2 (3.0)	2.6 (2.5)
Medications	4.3 (4.5)	5.8 (5.2)	6.9 (6.0)	7.8 (6.7)	9.6 (7.4)	11.8 (9.5)	17.1 (10.5)	5.3 (5.3)
Lab (individual tests)	25.2 (34.0)	35.8 (39.3)	46.3 (46.7)	59.5 (62.0)	80.6 (67.9)	109.8 (92.7)	162.0 (115.6)	33.7 (42.9)
Radiology	0.1 (0.9)	0.1 (1.1)	0.1 (1.0)	0.1 (0.8)	0.0 (0.1)	0.1 (0.9)	0.3 (2.0)	0.1 (1.0)

Radiology is outpatient radiology only. Numbers are rounded to 1 decimal place. Continuous variables expressed in mean (SD). Abbreviations: CKD – chronic kidney disease, ED – emergency department.

Table S7: Average annual healthcare use per person (mean, SD) – no PHC-linked

**Mean visits (SD)**	**At risk**, n = 10,670	**CKD 1**, n = 1,180	**CKD 2**, n = 658	**CKD 3a**, n = 953	**CKD 3b**, n = 434	**CKD 4**, n = 211	**CKD 5**, n = 97	**Overall**, n = 14,203
ED	0.7 (1.6)	0.8 (2.0)	0.9 (2.6)	0.8 (1.7)	1.1 (2.5)	1.3 (2.1)	1.8 (4.3)	0.7 (1.8)
Inpatient	1.0 (2.6)	0.9 (2.2)	1.2 (3.1)	1.3 (4.0)	1.5 (3.9)	4.0 (12.2)	16.8 (38.5)	1.2 (4.6)
→ Inpatient (dialysis only)	0.0 (1.2)	0.0 (0.4)	0.0 (0.7)	0.1 (3.1)	0.1 (2.2)	2.4 (10.9)	14.8 (36.5)	0.2 (3.8)
→ Inpatient (no dialysis)	1.0 (2.3)	0.9 (2.1)	1.2 (3.0)	1.2 (2.3)	1.4 (3.1)	1.6 (2.3)	2.1 (4.7)	1.0 (2.4)
Outpatient	3.2 (7.8)	1.8 (4.6)	3.3 (6.8)	4.2 (9.0)	3.8 (6.6)	6.2 (9.8)	7.6 (18.9)	3.2 (7.8)
Primary care	N/A	N/A	N/A	N/A	N/A	N/A	N/A	N/A
Medications	0.8 (2.5)	1.2 (2.8)	0.7 (2.4)	0.2 (1.4)	0.3 (1.8)	2.0 (5.9)	3.8 (7.4)	0.8 (2.6)
Lab (individual tests)	14.5 (33.0)	13.1 (27.2)	17.7 (41.2)	22.2 (52.9)	27.6 (52.4)	40.8 (64.1)	48.1 (86.4)	16.1 (36.9)
Radiology	0.1 (1.1)	0.1 (0.6)	0.1 (0.9)	0.1 (0.9)	0.0 (0.5)	0.0 (0.3)	0.0 (0.3)	0.1 (1.0)

Radiology is outpatient radiology only. Numbers are rounded to 1 decimal place. Continuous variables expressed in mean (SD). Abbreviations: CKD – chronic kidney disease, ED – emergency department.

Figure S2: Average annual total healthcare costs per person, by CKD stage – PHC-linked

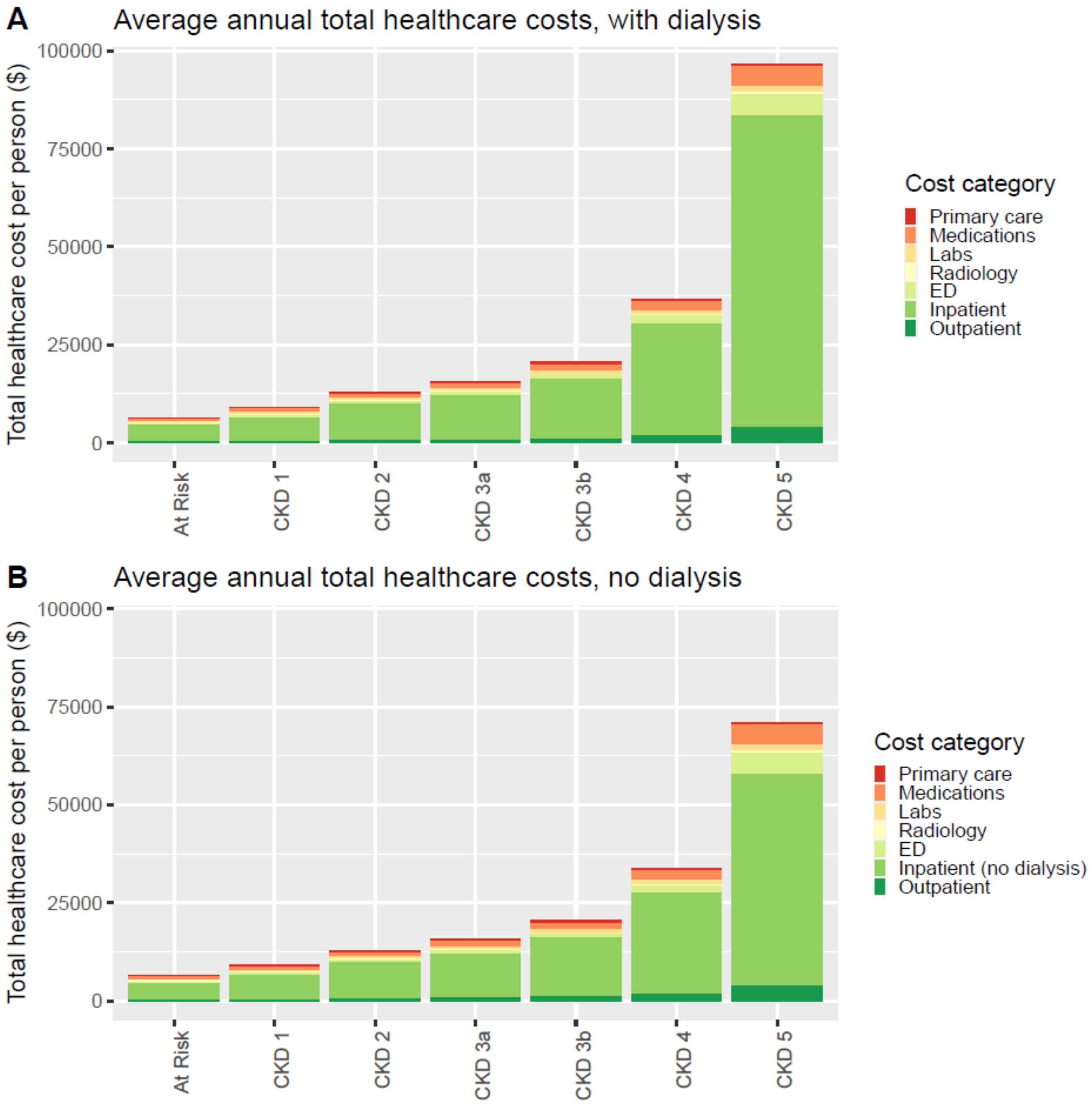

Figure S3: Average annual total healthcare costs per person, by CKD stage – no PHC-linked

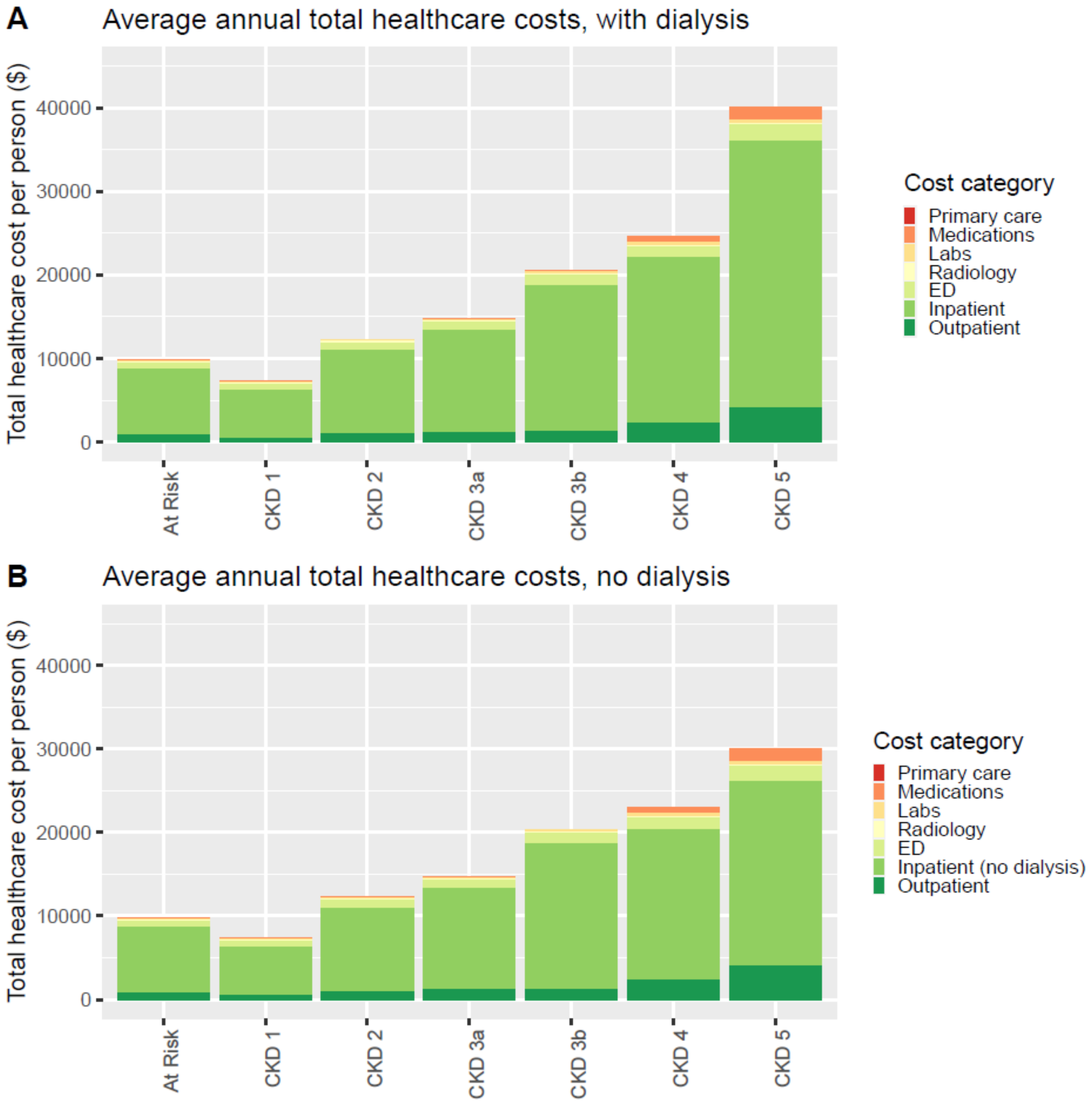

Table S8: Average annual healthcare use per person (mean, SD) – PHC-linked

**Mean cost (SD)**	**At risk**, n = 12,749	**CKD 1**, n = 6,265	**CKD 2**, n = 2,902	**CKD 3a**, N = 689	**CKD 3b**, n = 318	**CKD 4**, n = 183	**CKD 5**, n = 89	**Overall**, n = 23,195
ED	414 (1,410)	615 (1,533)	757 (1,678)	891 (2,113)	1,065 (2,009)	1,895 (2,932)	5,531 (9,689)	566 (1,680)
Inpatient	4,418 (19,317)	6,423 (20,847)	9,586 (29,549)	11,626 (38,481)	15,396 (37,182)	29,027 (54,348)	79,861 (89,738)	6,454 (24,048)
→ Inpatient (dialysis only)	4 (362)	0 (0)	57 (2,106)	28 (584)	140 (2,290)	3,044 (9,637)	25,682 (31,254)	135 (2,780)
→ Inpatient (no dialysis)	4,414 (19,298)	6,423 (20,847)	9,529 (29,351)	11,599 (38,454)	15,257 (36,887)	25,983 (51,767)	54,178 (80,861)	6,320 (23,562)
Outpatient	272 (939)	312 (1,162)	506 (1,531)	652 (1,410)	994 (1,540)	1,712 (2,363)	3,878 (8,766)	359 (1,283)
Primary care	301 (431)	392 (463)	551 (602)	619 (662)	709 (688)	671 (660)	603 (569)	376 (490)
Medications	744 (1,465)	957 (1,402)	1,032 (1,558)	1,354 (3,378)	1,605 (1,849)	2,327 (2,693)	5,091 (4,656)	897 (1,628)
Labs	252 (340)	358 (393)	463 (467)	595 (620)	806 (679)	1,098 (927)	1,620 (1,156)	337 (429)
Radiology	16 (163)	17 (170)	17 (173)	14 (134)	0 (3)	9 (55)	48 (335)	16 (165)
Total	6,417 (20,798)	9,073 (22,566)	12,912 (31,791)	15,752 (40,837)	20,574 (39,188)	36,739 (58,377)	96,632 (100,526)	9,004 (26,106)

Radiology is outpatient radiology only. $AUD 2023 dollars, rounded to whole dollars. Continuous variables expressed in mean (SD). Abbreviations: CKD – chronic kidney disease, ED – emergency department.

Table S9: Average annual total healthcare costs per person (mean, SD) – no PHC-linked

**Mean cost (SD)**	**At risk**, n = 10,670	**CKD 1**, n = 1,180	**CKD 2**, n = 658	**CKD 3a**, n = 953	**CKD 3b**, n = 434	**CKD 4**, n = 211	**CKD 5**, n = 97	**Overall**, n = 14,203
ED	695 (1,653)	683 (1,804)	898 (2,707)	900 (1,671)	1,193 (2,467)	1,348 (2,313)	1,857 (4,635)	750 (1,812)
Inpatient	7,978 (26,789)	5,901 (21,269)	10,206 (33,849)	12,376 (33,350)	17,652 (52,901)	19,927 (40,779)	32,166 (60,198)	8,842 (29,133)
→ Inpatient (dialysis only)	8 (805)	9 (324)	16 (412)	65 (2,005)	77 (1,604)	1,711 (7,876)	9,998 (24,893)	108 (2,583)
→ Inpatient (no dialysis)	7,970 (26,774)	5,892 (21,269)	10,190 (33,805)	12,311 (33,252)	17,575 (52,703)	18,216 (36,092)	22,167 (46,964)	8,734 (28,791)
Outpatient	839 (2,237)	487 (1,371)	925 (2,200)	1,149 (2,434)	1,229 (2,358)	2,282 (4,844)	4,036 (13,968)	889 (2,548)
Primary care	N/A	N/A	N/A	N/A	N/A	N/A	N/A	N/A
Medications	119 (972)	142 (509)	86 (417)	48 (561)	114 (705)	717 (2,248)	1,493 (3,643)	133 (979)
Labs	145 (330)	131 (272)	177 (412)	222 (529)	276 (524)	408 (641)	481 (864)	161 (369)
Radiology	17 (173)	10 (104)	16 (150)	16 (155)	9 (112)	1 (12)	2 (18)	16 (162)
Total	9,801 (28,620)	7,375 (23,255)	12,316 (36,401)	14,713 (35,483)	20,474 (55,059)	24,687 (45,516)	40,037 (72,630)	10,799 (31,290)

Radiology is outpatient radiology only. $AUD 2023 dollars, rounded to whole dollars. Continuous variables expressed in mean (SD). Abbreviations: CKD – chronic kidney disease, ED – emergency department.
