# Supplementary material for: Costs and healthcare use of patients with chronic kidney disease in the Northern Territory, Australia
Source: BMC Health Serv Res. 2024 Jul 9;24:791. doi: 10.1186/s12913-024-11258-8 (PMC11234693; doi:10.1186/s12913-024-11258-8)
Supplement: Supplementary file 2 — Supplementary Material 2. [file 12913_2024_11258_MOESM2_ESM.docx]

# Additional files 2 – Steering Committee authors

**Manuscript:** Costs and healthcare use of patients with chronic kidney disease in the Northern Territory, Australia

**Authors:** Winnie Chen, Kirsten Howard, Gillian Gorham, Asanga Abeyaratne, Yuejen Zhao, Oyelola Adegboye, Nadarajah Kangaharan, Mohammad Radwanur Rahman Talukder, Sean Taylor, Alan Cass, Territory Kidney Care Steering Committee

**Territory Kidney Care steering committee authors:**

In addition to named authors of the manuscript:

1. Andrew Bell – NT Department of Corporate and Digital Development
2. Bernie Cummins – NT Primary Health Network
3. Christine Connors – NT Health
4. Craig Castillon – NT Health
5. Debbie Gillon – Wurli Wurlinjang Health Service
6. Gautham Sundararaju – Sunrise Health Service Aboriginal Corporation
7. Heidi Faulkner – Katherine West Health Board Aboriginal Corporation
8. Jenny Jobst – Miwatj Health Aboriginal Corporation
9. John Boffa – Central Australian Aboriginal Congress
10. Karen Stringer – NT Health
11. Liz Moore – Aboriginal Medical Services Alliance Northern Territory
12. Lou Sanderson – Miwatj Health Aboriginal Corporation
13. Louise Maple-Brown – NT Health
14. Nathan Garrawurra – Miwatj Health Aboriginal Cooperation
15. Nathan Rosas – Wurli Wurlinjang Health Service
16. Margaret Cotter – Aboriginal Medical Services Alliance Northern Territory
17. Paul Burgess – NT Health
18. Paula Ferguson – NT Health
19. Pratish George – NT Health
20. Rama Nair – NT Cardiac Pty Ltd
21. Rebecca Bond – Sunrise Health Service Aboriginal Corporation
22. Ronald Ogilvie – Wurli Wurlinjang Health SErvice
23. Priyantha Wijesurendra – NT Health
24. Sam Heard – Central Australian Aboriginal Congress NT
25. Satpinder Daroch – NT Department of Corporate and Digital Development
26. Susan Clarke – Katherine West Health Board
27. Velma King – Wurli Wurlinjang Health Services
